# Trends of the main bibliometric indicators of *Anais Brasileiros de Dermatologia* (2010–2019)^☆^^☆☆^

**DOI:** 10.1016/j.abd.2020.11.006

**Published:** 2021-02-21

**Authors:** Hélio Amante Miot, Mayra Ianhez, Paulo Müller Ramos

**Affiliations:** a Departament of Dermatology, Faculty of Medicine, Universidade Estadual Paulista, Botucatu, SP, Brazil; b Department of Tropical Medicine and Dermatology, Instituto de Patologia Tropical e Saúde Pública, Universidade Federal de Goiás, Goiânia, GO, Brazil

**Keywords:** Bibliometrics, Impact factor, Journal article

## Abstract

**Background:**

The Anais Brasileiros de Dermatologia (*Anais Brasileiros de Dermatologia*) is the official journal of the Brazilian Society of Dermatology, which has been published since 1925, with free, bilingual access. There are several bibliometric indicators that estimate aspects of a journal’s scientific influence. Its analysis, based especially on the evolutionary trend, allows identifying the journal’s strengths and weaknesses, in addition to guiding editorial policies.

**Objectives:**

To evaluate the trends of the main bibliometric indicators of *Anais Brasileiros de Dermatologia*, in the decade of 2010–2019.

**Methods:**

Methodological study that proposed the analysis of bibliometric indicators published by Journal Citation Reports, SCImago, and Scopus, for the journal *Anais Brasileiros de Dermatologia*, from 2010 to 2019. The following aspects were evaluated: Impact factor, Immediacy index, SJR (SCImago), in addition to the number of citations, citable items, Cite Score, percentage of external citations, percentage of cited articles, percentage of citable articles, and the article influence score.

**Results:**

There was an increase in the main bibliometric indicators in the last decade: impact factor (0.337 to 1.121), immediacy index (0.054 to 0.204),*Eigenfactor* (0.00025 to 0.00394), SJR (0.176 to 0.453). The percentage of external citations (48.4% to 94.1%) and cited articles (24.7% to 51.4%) also increased. The increase in the impact factors of *Anais Brasileiros de Dermatologia* in the period was more significant than that of the dermatology journals (1,667 to 2,118) and the Brazilian journals (1,247 to 1,408), indexed in the Journal Citation Reports.

**Study limitations:**

Failure in the classification and registration of the databases.

**Conclusions:**

There has been a consistent increase in the scientific influence of *Anais Brasileiros de Dermatologia* in the last decade.

## Introduction

The journal Anais Brasileiros de Dermatologia, ABD (*Anais Brasileiros de Dermatologia,* ABD) is the official publication of the Brazilian Society of Dermatology and its purpose is to disseminate dermatological knowledge through original peer-reviewed articles, clinical and surgical trials, therapeutic research, new diagnostic techniques, and skin disease prevention. It has been regularly published since 1925, with free, bilingual access (Portuguese and English), bimonthly, being the only dermatological journal in Latin America indexed to the MEDLINE database.^1^

Bibliometrics has a branch called Scientometrics, which is dedicated to estimating the influence of a journal in the academic environment, evaluating different mathematical indices.^2^ The most disseminated bibliometric index is the Impact Factor (IF), which uses the citations from the Journal Citation Reports (JCR) as the database, available since 1975 on the Web of Science, part of the Science Citation Index (SCI), introduced in 1960 by the Institute for Scientific Information (ISI). This company, acquired by Thomson Reuters in 1992, was split up in 2016 to independently create Clarivate Analytics (https://clarivate.com/). Since then, this index has been considered one of the main methods to estimate the quality, importance, prestige, and influence of a scientific journal, when compared to others in the same area.^3^

The idea of quantifying the “impact” by counting citations led to the hierarchization of journals. However, several disadvantages have been indicated regarding the unrestricted use of the IF as the only metric for evaluating the quality of a scientific journal.^4^, ^5^ Considering that, new bibliometric indices were designed to meet the criticisms related to IF and in order to capture different aspects of the scientific influence of a journal, such as the Immediacy Index, the *Eigenfactor* Score, SJR (SCImago), the Cite Score, among others.^6^, ^7^

The possibility of comparing several bibliometric indices, especially from their temporal analysis, allows the identification of the journal's strengths and weaknesses, in addition to directing editorial policies.^8^

The aim of this study was to evaluate the trend of the main bibliometric indicators of ABD, in the decade of 2010 to 2019.

## Methods

This was a methodological study, which proposed analyzing the trend of the main bibliometric indicators published by *Journal of Citation Reports* (https://jcr.clarivate.com/JCRLandingPageAction.action), SCImago (http://www.scimagojr.com), and Scopus (https://www.scopus.com/sources), for the ABD journal (ISSN: 0365-0596), from 2010 to 2019.

Each bibliographic database divides scientific publications into citable and non-citable items, with slight differences between the sources. Overall, citable items include articles that contain an abstract, usually the original articles (such as investigations, case reports), and review articles. Congress abstracts, editorials, letters, amendments, book reviews, biographical items, and republications are usually considered non-citable items.^9^

The bibliometric indicators used in this study were the two-year IF (JCR), immediacy index, SJR (SCImago), in addition to the number of citations, citable items, Cite Score, percentage of external citations, percentage of cited articles, percentage of citable articles and the article influence score.^10^

The two-year IF is a metric based on the journal’s citations (numerator) in a given year, of articles published in the two previous years, divided by the total number of citable items published in the two previous years (denominator). It is calculated annually by Clarivate Analytics (formerly known as the Institute of Scientific Information - ISI), in the Journal Citation Reports.^10^

The immediacy index represents the average value of citations that a journal receives among the articles published in that same year, by journals from a bibliographic database.^10^

The *Eigenfactor* index uses the JCR database citation, similarly to the IF; however, it distributes weights to the journals that cite such articles, disregarding the self-citations (citations of references from the journal itself), and based on the five-year citation database.^10^

The SCImago scientific journal ranking (SJR) is an index based on the Scopus database.^10^ This indicator uses a method similar to the *Eigenfactor* index, based on the idea that citations from highly cited journals have greater weight than the ones that are seldom cited, without the influence of self-citations. The SJR derives its metric from a broader database (Scopus) and uses a period of three years for published articles. Although there are dozens of bibliometric indicators, the *Eigenfactor* index and SJR have been increasingly valued in recent years.^7^

The Cite Score is another metric that evaluates the list of citations per recently published article, calculating the citations of all items in relation to those published in the previous three years. Another difference from IF derives from the fact that the Cite Score includes all published items, including editorials and correspondence.^11^

The Article Influence Score is derived from the *Eigenfactor* index, which is divided by the citable items in a journal and scaled to an average of 1.0. It is based on a period of 5 years and also does not take self-citations into account.^10^

The median IF time series of dermatological and Brazilian journals indexed in the JCR were also evaluated, aiming to compare them with the ABD trend.

## Results

In 2019, ABD ranked 57^th^ out of 68 JCR journals in the “dermatology” category, and 978^th^ ​​out of 2,176 clinical medicine journals.

The ABD H index reached 39, indicating that there are at least 39 articles cited at least 39 times since the beginning of the citation registration by SCImago.

The most cited seven articles in 2019, published in 2017 and 2018, (according to the JCR) were all review texts, two related to infections (sporotrichosis, mucormycosis), two related to inflammatory diseases (Behçet and atopic dermatitis), two associated with cosmiatry (antioxidants and photoaging), and one about melanoma.^12^, ^13^, ^14^, ^15^, ^16^, ^17^, ^18^

Table 1 shows the values ​​of the main bibliometric indicators of ABD in the last decade. It is noteworthy the growth in indices related to article citations, such as IF, which increased 233%; the indicators of relevance within journals that cite ABD, such as the *Eigenfactor*, which increased 1,476%; or indicators linked to the contemporary nature of scientific information, such as the Immediacy Index, which increased 278%.

**Table 1:** Main bibliometric indicators of *Anais Brasileiros de Dermatologia* from 2010 to 2019.

Year	Impact factor	Immediacy Index	*Eigenfactor*	Citations	Citable items	SJR	Cite score	Percentage of external citations	Percentage of cited articles	Percentage of citable articles	Article influence score
2019	1.121	0.204	0.00394	2.585	108	0.453	1.900	94.1%	51.4%	84.6%	0.333
2018	1.050	0.125	0.00424	2.333	160	0.529	1.800	94.3%	52.8%	88.6%	0.320
2017	0.884	0.064	0.00342	1.847	219	0.520	1.800	93.0%	50.9%	91.9%	0.273
2016	0.978	0.048	0.00353	1.456	146	0.478	1.800	92.0%	55.1%	94.1%	0.260
2015	0.880	0.074	0.00344	1.282	204	0.516	1.600	87.8%	55.7%	95.1%	0.261
2014	0.723	0.130	0.00237	1.077	154	0.404	1.500	76.2%	47.9%	94.6%	0.185
2013	0.866	0.048	0.00175	867	228	0.484	1.400	71.8%	49.9%	92.8%	0.161
2012	0.618	0.115	0.00117	656	122	0.396	1.000	71.2%	42.4%	90.9%	NA
2011	0.554	0.043	0.00047	575	235	0.211	0.500	50.4%	37.1%	88.9%	NA
2010	0.337	0.054	0.00025	366	129	0.176	0.300	48.4%	24.7%	87.0%	NA

**Figure 1:**
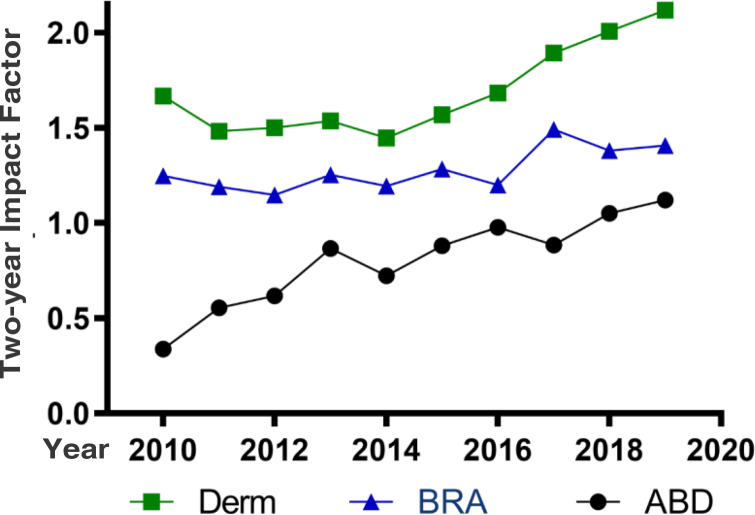
Time series of the two-year Impact Factor of *Anais Brasileiros de Dermatologia* (ABD, Anais Brasileiros de Dermatologia), and the medians of the impact factors of the journals that comprise the dermatology database (Derm), and the Brazilian journals (BRA), according to the Journal of Citation Reports in the period from 2010 to 2019.

Fig. 1 shows the evolution (2010–2019) of the two-year IF of ABD, of the journals that comprise the dermatology database, and of the Brazilian journals, according to the JCR database. The ABD showed, in the period, an IF evolution that was superior to the other dermatology journals (a 27% increase), or even of the other Brazilian journals (13% increase).

The time period from article approval to the online publication of the two review articles, of the last 2019 ABD issue, was two and four months. Of the eight investigation articles, the average time period between approval and online publication was 8 months, with a potential loss to the possible number of citations.

## Discussion

The analysis of the main bibliometric indicators of the ABD showed a substantial and consistent increase in the scientific influence and contemporaneity of its publications, in addition to the fact that their trends suggest the maintenance of growth in the following years.

The evolution of the ABD IF, in the last decade, was more significant than the pool of dermatology journals, as well as the Brazilian scientific journals indexed in the JCR database. This increase may also have been a result of the search for publications of higher-quality research, usually directed to foreign journals with a higher IF, since academic evaluations, positions to work as civil servants, scientific funding, or even the categorization of postgraduate courses are strongly determined by the IF of the journals in which the articles are published. However, it should be noted that the use of IF as the only scientific quality metric has serious restrictions.^19^, ^20^, ^21^

IF is influenced by the fraction of citable items in a journal, since the non-citable items are added to the IF numerator, when they are cited, but do not influence their denominator. Several dermatology journals have strategically reformatted some sections of the journals (*e.g.*, case reports and brief communications) into letters, aiming to allow a greater volume of publications without damaging the IF.^9^, ^22^ In 2014, ABD recorded 154 (94.6%) citable items, which was reduced to 108 (84.6%) in 2019, largely due to the introduction of the “case letter” and “research letter” sections to include case reports and brief communications. The interruption of the publication of case reports (approved and held for more than a year), should significantly impact the growth of the IF in the next 2 years. The journal’s H index, especially if calculated based on articles published in the last 5 years, is an alternative to bypass IF bias caused by non-citable items.^23^

The composition of citable items in journals can also impact the IF. Overall, review articles, large clinical trials, articles on common diseases (*e.g*., acne), or of international social relevance (*e.g*., COVID-19) are more cited than case reports, or articles of local relevance (*e.g*., lobomycosis).^24^, ^25^ In 2019, all the most cited articles from ABD were review articles. However, the latency of more than 6 months between the approval of investigation articles and their online publication (ahead of print) may be harming the recent citations of these categories in ABD. According to the SCImago database, the IF of cited articles in three years for ABD is 17.5% higher than the IF based on two years.

In addition to the rapid online publication of approved articles, which directly interests the most productive researchers, another factor that can attract better quality research is the restriction regarding the number of authors, which has limits in ABD. However research teams, especially in multidisciplinary projects, are expanding their staff worldwide, which requires a higher number of authors in scientific communications.^26^ Although certain groups inflate the number of authors aiming to generate fraudulent academic scores, ABD must develop mechanisms to recognize true contributions from multiple authors.^27^

IF performance suffers the consequences of self-citations, which can inflate the number of citations, with the incentive to self-citations being considered editorial malpractice.^22^, ^28^ As an alternative, JCR has an IF measure that disregards self-citations, in addition to indices such as *Eigenfactor* and SJR, which do not use them in their estimates. ABD has increased external citations from 48% to 94% in the last decade, resulting in a self-citation rate of less than 10%. This represents a progressive and greater repercussion of its articles in the scientific community and indicates the consistency of the editorial policy.

Another practice that artificially inflates IF is the cross-citation agreement between journals (stacking). Several scientific journals have been penalized by the JCR due to suspected stacking.^29^ Indicators that score the relevance of journals that cite articles, such as the *Eigenfactor*, SJR, and Article Influence Score, are less affected by stacking than IF. The increase in the values ​​of all these indicators for ABD, in the last decade, demonstrates the gain of influence with the international scientific community.

The manuscripts submitted electronically to ABD are peer-reviewed, consisting of specialists from different areas of dermatological knowledge, who do it anonymously. Quantitative investigation articles are previously submitted to the analysis of methodology and results by statisticians. This editorial process increases the final quality of the published articles, as well as providing authors with critical reflections on their own research.^30^ It is, therefore, crucial to maintain a homogeneous body of reviewers, to promote methodological training and alignment with the journal editorial policies, to guarantee the quality and increase the influence of ABD in different areas of knowledge.^31^

The journals that comprise the main bibliographic databases consist mainly of journals written in the English language, which maximizes the citation of articles by English-speaking researchers.^32^ The bilingual publication of ABD favors the reading and citation of its articles by researchers from different countries.^33^ Moreover, free access to the full texts of ABD through the SciELO (https://www.scielo.br/) and Pubmed Central (https://www.ncbi.nlm.nih.gov/pmc/) platforms allows unrestricted dissemination of scientific information, democratizing knowledge to researchers with limited resources, as well as attracting higher-quality research.^34^, ^35^ Also, the Brazilian Society of Dermatology subsidy for the submission, statistical review, and publication of articles in ABD, maximizes publication opportunities for authors and institutions with fewer research resources, a model of good practice for scientific publications.^36^

The main limitation of this study is the fact that it is based on bibliometric indicators subject to classification and registration failures, in addition to the impossibility of covering all existing indicators. The items considered citable in the different bibliometric databases show significant differences. In 2019, while the JCR considered 108 citable articles for ABD, Scopus computed 182 items, which influences the denominator of the impact factor calculation.^9^

The incorrect recording of the bibliographic references of the articles was pointed out as an important element in the automated citation weighting, for instance, the JCR annually computes millions of citations coming from tens of thousands of journals.^37^ Errors in the spelling of the authors’ names, titles of articles, and nomenclature of journals are factors that hinder the registration of citations.

Citations from different sources also differ depending on the journals included in their database; the more journals they include, the higher the probability of having citations from ABD. For instance, the JCR dermatology database, in 2019, consisted of 68 journals, whereas SCImago comprised 123 journals. In parallel, the two-year IF of ABD in the SCImago database is 1.221, while in the JCR database, it is 1.121. Despite the differences in values, the indicators based on the number of citations correlate quite well with each other. It should be noted that not only do dermatological journals cite ABD, but they are certainly the largest source of ABD citations.

Similarly, there is a tendency toward an annual increase in the number of journals in the databases, which has the potential to inflate, by itself, the overall impact of the journals. Only in the dermatology section, in 2019, JCR showed a 23.6% increase in its journal database, when compared to 2010.

Bibliometric indicators should be carefully analyzed when assessing the impact factor of a journal. Articles of local importance, such as outbreaks of regional diseases, or very rare diseases, can be extremely valuable but are less likely to be cited. Also, journals from different areas of knowledge show characteristic citation flows, which makes it difficult to compare journals on different topics. The median IF of dermatology journals in 2019 was 2,118, while it was 3.135 for rheumatology, and 3.477 for allergy/immunology. To make it clearer, social science journals are less cited, or cited later, than medical journals, in addition to the fact that their reference database significantly depends on books, which are not indexed as citable items in the main databases, impairing their comparability by IF.^19^, ^24^, ^38^

Finally, the main bibliometric indicators refer to the scientific influence of a journal and are not independently related to any article. Journals with a high IF publish articles that will never be cited, and, similarly, articles with great scientific impact are published in less important journals.^8^

Researchers should prioritize the publication of full articles, with broad results and discussion sections, rather than splitting the results into different articles (salami science), to inflate their own metrics.^39^ The purpose of a scientific journal, as a vehicle for communication and criticism of the established scientific paradigm, must prevail over the choice and acceptance of an article due to its citation potential.^3^, ^22^

## Conclusion

There has been a consistent increase in the main bibliometric indicators of ABD in the last decade, especially when compared to the indices of global dermatological journals and national medical scientific journals. We highlight the increase in the rate of citations of articles, its open access, it being a bilingual publication, an editorial policy involving a high number of citable articles (maintaining the flow of case reports), and peer review, guaranteeing a better final quality for the article. Despite the substantial growth of ABD, the overestimation of bibliometric indices by funding agencies and universities continues to divert submissions to ABD, compared to international journals with a higher IF.

## Financial support

None declared.

## Authors’ contributions

Hélio Amante Miot: Study conception; data analysis; writing, review, and approval of the final version of the manuscript.

Mayra Ianhez: Study conception; writing, review, and approval of the final version of the manuscript.

Paulo Müller Ramos: Study conception; writing, review, and approval of the final version of the manuscript.

## Conflicts of interest

None declared.
